# Supplementary material for: Solute trapping and non-equilibrium microstructure during rapid solidification of additive manufacturing
Source: Nat Commun. 2023 Dec 2;14:7990. doi: 10.1038/s41467-023-43563-x (PMC10693635; doi:10.1038/s41467-023-43563-x)
Supplement: Supplementary file 1 — Supplementary information [file 41467_2023_43563_MOESM1_ESM.docx]

**Supplementary Information**

**Solute trapping and non-equilibrium microstructure during rapid solidification of additive manufacturing**

Neng Ren^1^, Jun Li^1,*^, Ruiyao Zhang^2^, Chinnapat Panwisawas^3,*^,Mingxu Xia^1^, Hongbiao Dong^4^, Jianguo Li^1^

^1^Shanghai Key Laboratory of Advanced High-temperature Materials and Precision Forming, School of Material Science and Engineering, Shanghai Jiao Tong University, Shanghai 200240 P.R. China.

^2^Centre of Excellence for Advanced Materials, Dongguan, 523808, China

^3^School of Engineering and Materials Science, Queen Mary University of London, London E1 4NS, United Kingdom.

^4^School of Engineering, University of Leicester, Leicester LE1 7RH, United Kingdom.

^*^Corresponding author: Jun Li, e-mail: [li.jun@sjtu.edu.cn](mailto:li.jun@sjtu.edu.cn), <https://orcid.org/0000-0003-4915-0336> and

Chinnapat Panwisawas, e-mail: [c.panwisawas@qmul.ac.uk](mailto:c.panwisawas@qmul.ac.uk), <https://orcid.org/0000-0003-2141-5865>

**Supplementary Methods**

## Model description on the alloy system

The solidification path of superalloys during rapid heating and cooling in additive manufacturing (AM) is rather complicated. Cellular/dendrites (γ primary phase), MC carbides, borides, eutectic structures, and even the precipitation of γ′ phase can be observed in the as-additively manufactured structure. Under the consideration of the model compatibility and the uncertainty of the growth dynamics of the intermetallics, the proposed model only describes the morphological evolution of the primary γ phase, while the formation of compounds such as carbides and borides, is not considered in the model framework. Upon cooling after the local temperature drops to eutectic temperature, it is assumed that the residual liquid in the intercellular/interdendritic region should all transform to solid eutectic phases.

Due to the complicated nature of the solute diffusion and solute partition of the multicomponent system, superalloys are simplified to linearised equivalent binary systems. For each binary system, the molten metal is treated as dilute solutions, and the effects including chemical activities, chemical reactions, element interdiffusion, and the change in solidification path are neglected. The equivalent solute, the corresponding equivalent partition coefficient, liquidus temperature slope, and solutal expansion coefficient are calculated based on the following equations:^1,2^

| $\bar{c}_{0}=\sum_{i=1}^{N-1} c_{0_{i}}$ |  |
| --- | --- |
| $\bar{c}_{0}\cdot\bar{m}=\sum_{i=1}^{N-1} m_{i}c_{0_{i}}$ |  |
| $\bar{c}_{0}\cdot\bar{m}/\bar{k}=\sum_{i=1}^{N-1} m_{i}c_{0_{i}}/k_{i}$ |  |
| $\bar{c}_{0}\cdot(1-\bar{k})\cdot\bar{\beta}_{\text{C}}=\sum_{i=1}^{N-1} c_{i}(1-k_{i})\beta_{\text{C}_{i}}$ |  |

where the subscript *i* stands for the phase diagram parameters of the *i*^th^ Ni-X systems in the multicomponent superalloys, and *N* is the number of the considered elements. X represents the solute elements which are generally added to the nickel-based superalloys, e.g., Al, Co, Cr, Fe, Mo, Ta, Ti, W, Re, Nb, Hf, C, B, and Zr. The partition coefficients, the liquidus slopes, and the solutal expansion coefficients of the linearised binary Ni-X systems in the superalloys of CM247LC, Inconel 718 and ABD-850AM are listed in Supplementary Table 1. The partition coefficients of the solute elements in CM247LC and Inconel 718 were derived from the references, while the ones in ABD-850AM were calculated using the method proposed by Hobbes et al^3^ (Eq. 5):

| $k=a+\sum_{i=1}^{n} b_{i}C_{i}$ |  |
| --- | --- |

where *a* and *b* are regression coefficients, *C* is the nominal concentration of the solute element *i* in the superalloys.

Supplementary Table 2 lists the coefficients of the regressed equations used to calculated the partition coefficients of Al, Cr, Co, Mo, Ta, Ti, and W. It should be noted that the equation for the partition coefficient of Ti was not reported, and it was derived using multiple linear regression analysis. The liquidus slopes of the elements were deduced from the corresponding binary Ni-X phase-diagrams^4^. Besides, the solutal partition coefficients of Zr and C in the Ni-Zr and Ni-C systems are 0.06 and 0.34^5,6^, respectively. Correspondingly, the liquidus slopes are -21.51 K wt.%^-1^ and -61.17 K wt.% ^-1^. The values which were not listed in the tables indicate that the corresponding factors have not been considered in the simulations.

**Supplementary Table 1**

Phase diagram parameters of the Ni-X systems (X for the listed solute elements) in the superalloys of CM247LC, Inconel 718, and ABD-850AM.

| Elements | Partition coefficient | | | Liquidus slope  K wt.% ^-1^ | Solutal expansion coefficient wt.% ^-1^ |
| --- | --- | --- | --- | --- | --- |
|  | CM247LC | Inconel 718 | ABD850-AM |  |  |
| Al | 0.92 | 1.03 | 0.98 | -5.35 | 2.50×10^-2^ |
| Co | 1.05 | -- | 0.94 | 0.01 | 1.13×10^-2^ |
| Cr | 1.00 | 0.82 | 0.77 | -2.15 | 1.90×10^-3^ |
| Fe | 1.00 | 1.2 | -- | -0.53 | -- |
| Hf | 0.12 | -- | -- | -8.82 | -- |
| Mo | 0.72 | 0.65 | 0.67 | -5.72 | -1.80×10^-3^ |
| Ta | 0.71 | -- | 0.70 | -5.00 | -4.60×10^-3^ |
| Ti | 0.74 | 0.41 | 0.57 | -16.30 | 8.20×10^-3^ |
| W | 1.23 | -- | 0.51 | 2.45 | 5.30×10^-3^ |
| Nb | -- | 0.48 | 0.48 | -10.50 | 8.00×10^-4^ |

**Supplementary Table 2**

Models of partition coefficients under the effect of elemental interactions (proposed by Hobbs et al^3^).

| *k_i_* | *a* | *b* [Al] | *b* [Cr] | *b* [Co] | *b* [Mo] | *b* [Ta] | *b* [W] |
| --- | --- | --- | --- | --- | --- | --- | --- |
| Al | 0.960 | 0.0028 | 0.00262 | 0.00589 | -- | -- | -0.00846 |
| Cr | 0.779 | 0.0416 | 0.0015 | -0.00506 | -0.00116 | 0.00791 | -- |
| Co | 0.970 | 0.0119 | -- | -0.00312 | -0.00112 | 0.00356 | 0.00302 |
| Mo | 1.10 | 0.0168 | -- | -- | -0.011 | -0.0129 | -- |
| Ta | 0.704 | 0.0168 | 0.00525 | -0.00388 | 0.00432 | 0.0109 | -0.0136 |
| Ti | 0.4863 | 0.0415 | -0.0014 | 0.0071 | -0.0351 | -0.0066 | -- |
| W | 0.281 | 0.0988 | -0.00316 | 0.0101 | -0.0063 | 0.0289 | -0.00325 |

According to the fundamental of solidification, the solute concentration can be considered to range from *kC*_0_ to *C*_0_/*k*. Correspondingly, the *SI* ranges from *k*-1 to 1/*k*-1 for the case where *k*<1. The *SI* of a certain element can be reproduced based on the assumption that the solute partition is independent from the other solute elements. Thus, the *SI* of the solute element *i* can be calculated with:

| $SI_{i}=\begin{matrix} SI_{\text{e}}\cdot\left(k_{i}-1\right)/\left(\bar{k}-1\right) & SI<0 \\ SI_{\text{e}}\cdot\left(1/k_{i}-1\right)/\left(1/\bar{k}-1\right) & SI\geq0 \end{matrix}$ |  |
| --- | --- |

where *SI*_e_ is the segregation index of the equivalent solute element. The segregation (solute mass fraction) of the key solute elements in the superalloys, such as Hf in CM247LC and Nb in Inconel 718, can be deduced from the segregation distribution of the equivalent binary system.

## Simulation parameters

The thermophysical properties of the superalloys CM247LC, Inconel 718, and ABD-850AM are listed in Supplementary Table 2. In the benchmark cases (Inconel 718), we used the same process parameters as the reference^7^ for the validation of the simulation results. The process parameters are listed in Table 2. The powder distribution is determined using discrete element method^8^, as shown in Supplementary Figure 1. The two-dimensional computational domain and powder distribution are extracted from the longitudinal section perpendicular to the scanning path to represent the real powder distribution.


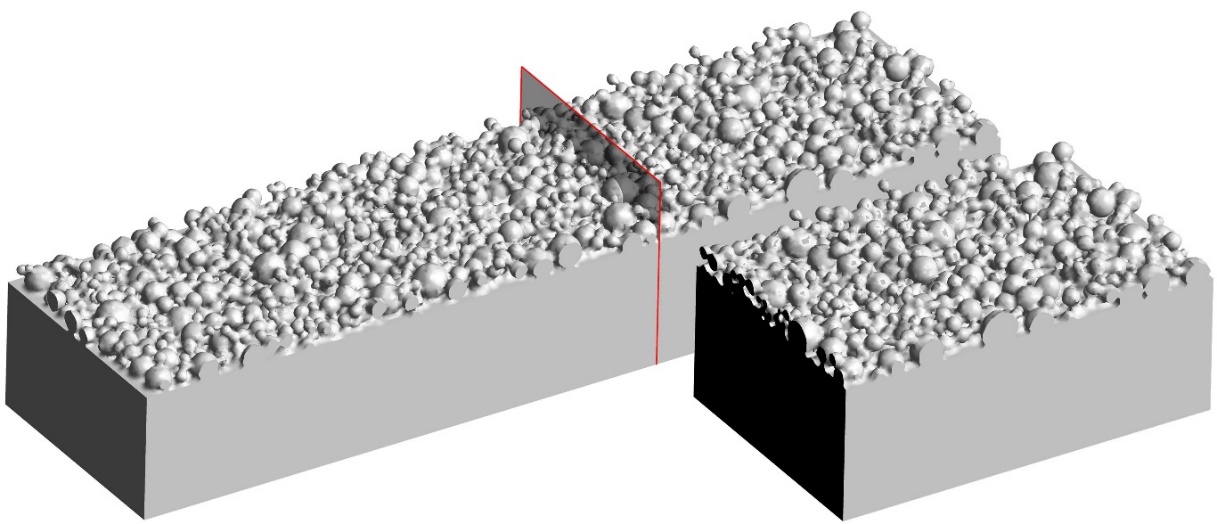


**Supplementary Figure 1.** Three-dimensional particle distribution of the powder bed and the two-dimensional computational domain (including the powder and the substrate), which is extracted from the longitudinal section.

**Supplementary Table 2**

Thermophysical properties of the superalloys CM247LC, Inconel 718 and ABD-850AM^7,9–13^.

| Properties | CM247LC | Inconel 718 | ABD850-AM |
| --- | --- | --- | --- |
| Density (kg∙m^-3^) | 7470 | 7578 | 7610 |
| Thermal conductivity (W∙m^-1^∙K^-1^) | 26.6 | 29 | 30 |
| Specific heat (J∙kg^-1^∙K^-1^) | 580 | 720 | 690 |
| Latent heat of fusion (J kg^-1^) | 264,000 | 210,000 | 262,930 |
| Boiling temperature (K) | 2940 | 3005 | 2950 |
| Latent heat of evaporation (J kg^-1^) | 6.70×10^6^ | 6.31×10^6^ | 6.70×10^6^ |
| Molar mass of metal vapor (g∙mol^-1^) | 59.75 | 59.75 | 59.75 |
| Dynamic viscosity (Pa∙s) | 7.33×10^-3^ | 7.2×10^-3^ | 7.57×10^-3^ |
| Surface tension coefficient (N∙m^-1^∙K^-1^) | -4.0×10^-4^ | -1.0×10^-4^ | -6.09×10^-4^ |
| Temperature sensitivity of surface tension coefficient (N∙m^-1^) | 1.45 | 1.882 | 1.813 |
| Thermal expansion coefficient (K^-1^) | -2.14×10^-4^ | -1.6×10^-5^ | 2.8×10^-5^ |
| Solute diffusion coefficient in liquid (m^2^∙s^-1^) | 4.93×10^-9^ | 3.0×10^-9^ | 1.06×10^-9^ |
| Solute diffusion coefficient in solid (m^2^∙s^-1^) | 2.5×10^-13^ | 1.0×10^-12^ | 3.0×10^-12^ |
| Maximum nuclei density (m^-3^) | 10^15^ | 10^15^ | 10^15^ |
| Mean nucleation undercooling (K) | 21.0 | 9.5 | 10.1 |
| Standard deviation of Gaussian distribution (K) | 2.0 | 2.0 | 2.0 |

Pressure-implicit with splitting of operators (PISO) algorithm was used to solve the pressure-velocity coupling governing equations. The source terms and auxiliary variables were updated at the beginning of each iteration. The governing equations were solved implicitly with a convergence criterion of 10^-3^ for the mass, momentum, and solute equations, and 10^-6^ for the energy equation. Up to 20 iterations were performed in every time step to reduce the residuals of the solutions of the governing equations. For the solution of the multiphase flow of high flow velocity with such a small grid size, a small timestep of 5 ns was set to ensure the global Courant number was less than 1.

**Supplementary Results and Discussion**

## Quantitative analysis on the convective contributions

According to the fundamental solidification^14^, an analytical Gulliver-Scheil model is proposed to describe the effect of melt convection on the solute profile parallel to the crystal growth direction. Supplementary Figure 2a show the schematic diagram of the employed analytical Gulliver-Scheil model. Under the “sufficient diffusion in liquid” condition, the solute in the front of the solid/liquid interface could be well mixed with the melt by the strong thermal-fluid melt flow. In contrast, under the ‘no convection’ condition, there is a solute boundary layer at the solidification front. Melt flow intensity determines the thickness of solute boundary and the solute concentration at the solid/liquid interface. However, under the rapid crystal growth condition, the solute boundary layers can also be suppressed due to the solute trapping effect, as shown in Supplementary Figure 2b.

The analytical model is then employed to estimate the contribution of the two factors. Assuming the solid/liquid interface could reach liquidus (*T*_liq_) all the time, such as from time *n* to *n*+1, cooling rate (*Ṫ*) can be regarded as the increase in crystal growth undercooling degree (Δ*T*) in a unit time (Δ*t*), as shown in Eq. 7. At the solute boundary layer, the interfacial solute concentration (*C*_l_) equals to the division of the initial concentration (*C*_0_) and the partition coefficient (*k*). And the interfacial velocity (*V*) is calculated using Lipton-Glicksman-Kurz (LGK) model, as shown in Eq. 8 (where *α*_1_, *α*_2_, and *α*_3_ are fitted growth kinetic constants^15^). Then the partition coefficient can be calculated based on the rapid solidification model established by Aziz et al^16^, as shown in Eq. 9, where *V*_d_ is critical interfacial velocity and *k*_e_ is equilibrium partition coefficient. The non-equilibrium partition coefficient can be used to evaluate microstructure and solute profile. Based on Eq. 7, 8, and 9, iterations were conducted to achieve the solution of the variables.

Supplementary Figure 2c demonstrates the comparison of the two cases and quantifies the contributions of melt convection under the evolution of crystal growth undercooling degrees. Here, the crystal growth undercooling degree on the *x*-axis is the one in the no convection case. For the Nb element in Inconel 718, under a relative low cooling rate (close to the equilibrium state), the melt convection can provide extra crystal growth undercooling degree as large as about 57 K. During the same thermal history, this promotes the solute trapping effects and can raise the partition coefficient from 0.48 to as high as 0.7.

|  |  |
| --- | --- |
| $V\left(\Delta T\right)=\alpha_{1}\Delta T+\alpha_{2}\Delta T^{2}+\alpha_{3}\Delta T^{3}$ |  |
| $\begin{matrix} k=\frac{k_{\text{e}}+V/V_{\text{d}}}{1+V/V_{\text{d}}} & C_{\text{l}}=C_{0}/k & \end{matrix}$ |  |

To further illustrate the contribution of melt convection, comparing a representative simulation results without convection^17^ and the experimental data^18^ , the simulated (quasi) solute trapping region is about half the height (5 μm in difference) of the experimentally measured one under similar operating conditions. The differences can also quantitatively indicate the convective contributions to the evolution of microstructure and solute transport.


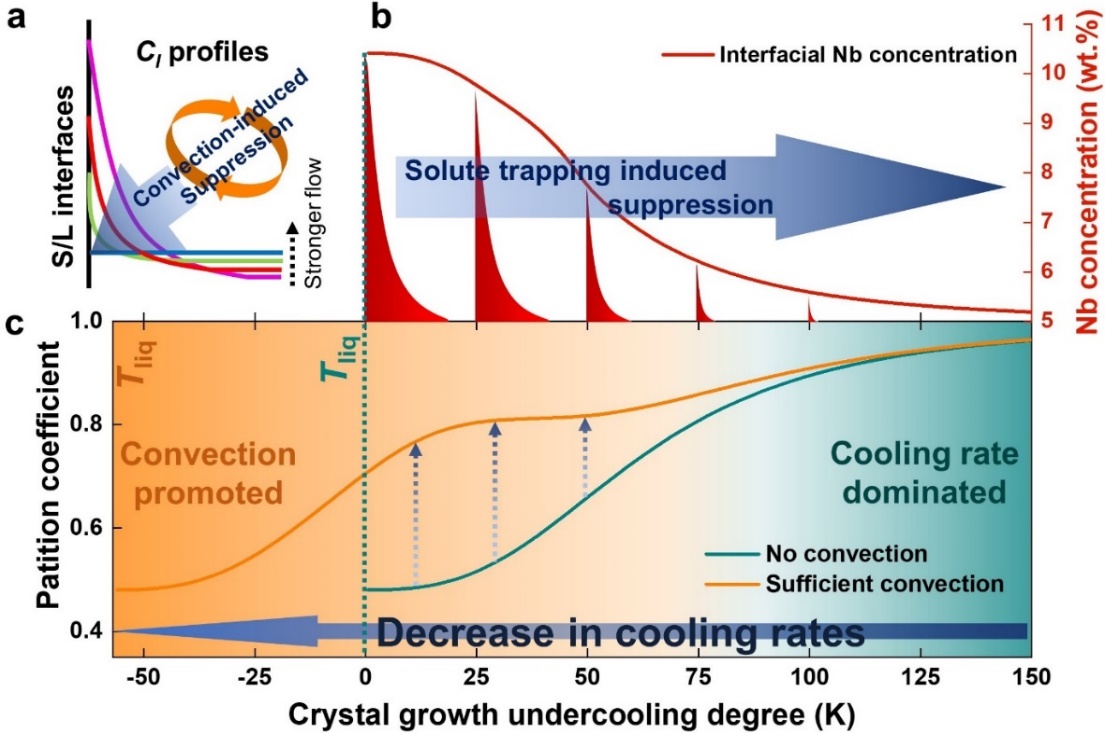


**Supplementary Figure 2** Effect of melt convection on the solidification behaviours based on the analytical Gulliver-Scheil model. **a** Schematic diagram of solute boundary layer at the solidification front. **b** The solute profiles under different crystal growth conditions. **c** Convective contribution to the solute trapping effect under different crystal growth conditions.

## Extended analysis of the three-dimensional case

According to the reported three-dimensional flow field analysis^7,19^ on the melt pool of LPBF process, the melt flow parallel to the scanning direction would scour the solidification front at the bottom of the melt pool at the tail, transferring the solute elements rejected by the cellular (dendritic) trunks to the forward of the melt pool of higher temperature. The melt pool profile is much longer in the scanning direction^20^. The melt pool would be more stable with less swaying on the perpendicular section in the real 3D case. Consequently, there would be less transitions and unexpected evolution in the solute distribution and microstructure induced by the perturbation of melt flow. More importantly, the local heat in the tail of melt pool could still be compensated by the forward high-temperature melt even after the laser spot has passed away, which significantly prolongs the solidification process and reduces the cooling rate. This effect leads to a wider gap in approaching the (quasi) solute trapping non-equilibrium state.

## Process conditions in the cited experiments

It should be noted that the experimental conditions for the results shown in Fig. 3 c, f, and i are slightly different from the benchmark case. Here are supplementary instructions on the experimental details. In the experiments conducted by Tang et al.^21^ to print CM247LC and ABD-850AM (the results of which are shown in Fig. 3c and i), a modulated laser was used to perform point-to-point scans, and a raster scan with 67° rotation for each layer was performed. The process parameters used were: laser power 200 W, layer thickness 30 μm, hatch spacing 50 μm, point distance 90 μm, and exposure time at each point 50 μs. In the experiments performed by Lee et al.^18^ to LPBF Inconel 718 (see Fig. 3f for the experimental results), a laser power of 200 W, a scanning speed of 1000 mm/s, a hatch spacing of 0.1 mm, a powder layer thickness of 20 μm, and a particle size range of 10 to 50 μm were used.

**Supplementary Figures**


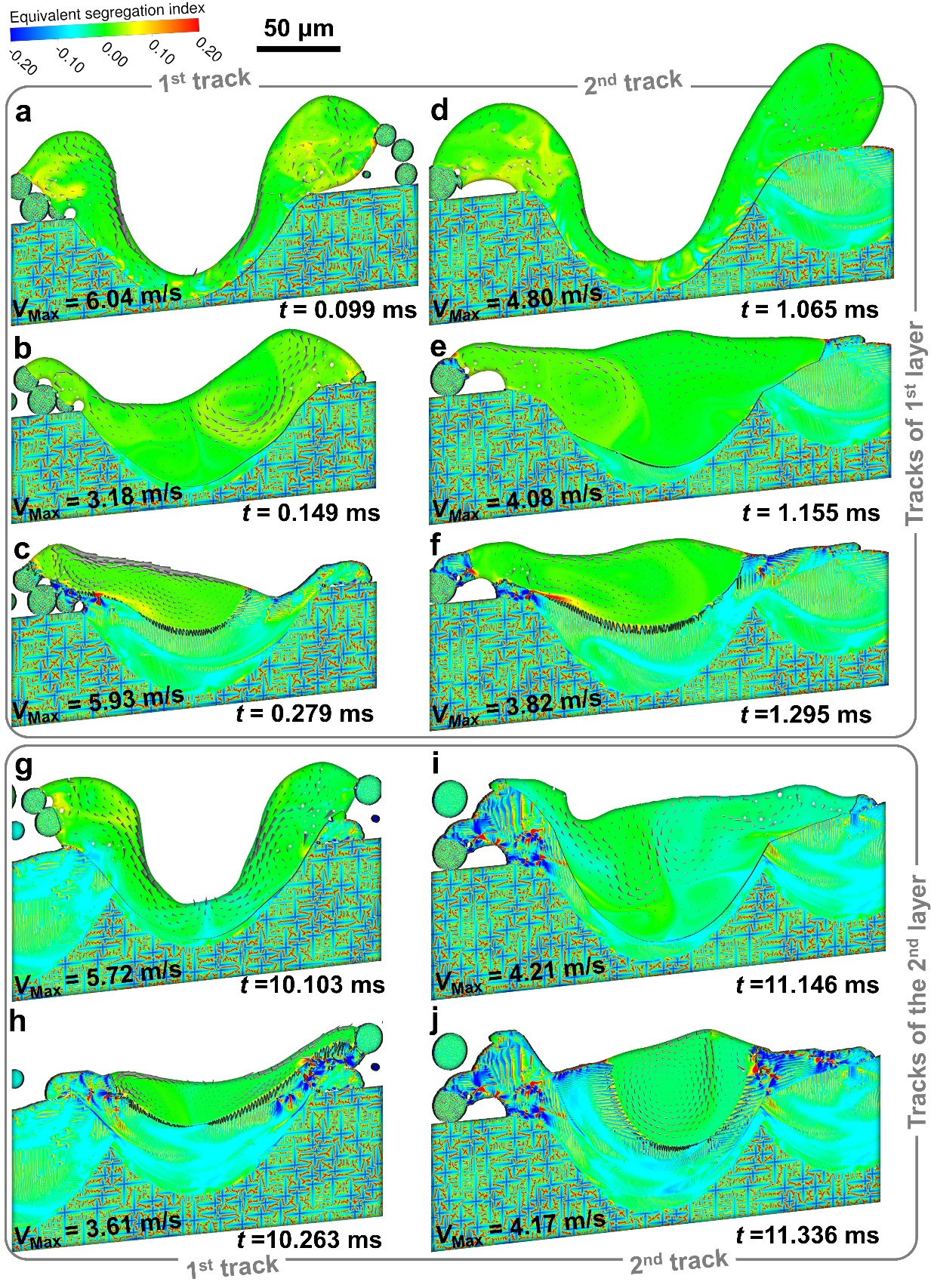


**Supplementary Figure 3.** Close-up views of flow field and solute profile of metal pools during the melting-solidification process of laser powder bed fusion of ABD-850AM.


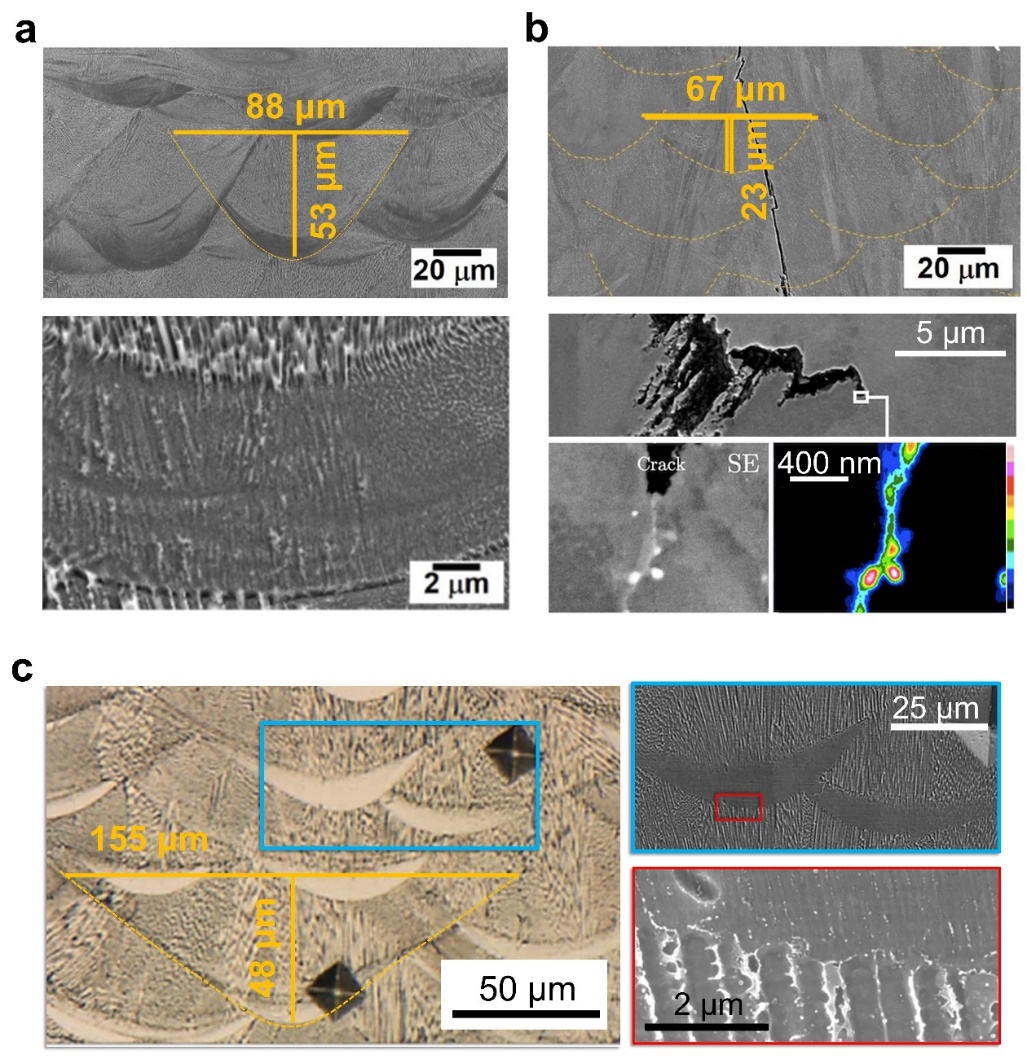


**Supplementary Figure 4.** Close-up views of the melt pools and characteristics of the as-printed microstructures observed in Fig. 3c, 3i, and 3f. **a** Dimensions of melt pools and solute trapping regions, and microstructural characteristics of ABD-850AM observed by Tang et al.^21^ **b** Grain structure, solidification cracks, and normalised Hf distribution in the LPBF-ed CM247LC observed by Ghoussoub et al.^22^ **c** Morphology and size of the solute trapping regions, and microstructural features around the solute trapping region of printed Inconel 718 observed by Yi et al.^18^

*Images were rescaled to improve readability.

**The scale bar of the original figure in Ref. 18 (Fig. 4e) should be nanometre rather than micrometre.

**Supplementary References**

1. Zhang, H. & Xu, Q. Multi-scale simulation of directional dendrites growth in superalloys. *J. Mater. Process. Technol.* **238**, 132–141 (2016).

2. Yang, B. J., Stefanescu, D. M. & Leon-Torres, J. Modeling of microstructural evolution with tracking of equiaxed grain movement for multicomponent Al-Si alloy. *Metall. Mater. Trans. A* **32**, 3065–3076 (2001).

3. Hobbs, R. A., Tin, S. & Rae, C. M. F. A castability model based on elemental solid-liquid partitioning in advanced nickel-base single-crystal superalloys. *Metall. Mater. Trans. A* **36**, 2761–2773 (2005).

4. Reed, C. R. *The Superalloys: Fundamentals and Applications*. (Cambridge University Press, 2006).

5. Zeisler-Mashl K L, P. B. J. Segregation During Solidification in the MAR-M247 System. in *Superalloys 1992* 175–184 (Minerals Metals & Materials Society, 1992). doi:10.7449/1992/Superalloys_1992_175_184.

6. Taha, M. A. & Kurz, W. About Microsegregation of Nickel Base Superalloys. *Int. J. Mater. Res.* **72**, 546–549 (1981).

7. Lee, Y. S. & Zhang, W. Modeling of heat transfer, fluid flow and solidification microstructure of nickel-base superalloy fabricated by laser powder bed fusion. *Addit. Manuf.* **12**, 178–188 (2016).

8. Panwisawas, C. *et al.* Mesoscale modelling of selective laser melting: Thermal fluid dynamics and microstructural evolution. *Comput. Mater. Sci.* **126**, 479–490 (2017).

9. Yu, Y., Li, Y., Lin, F. & Yan, W. A multi-grid Cellular Automaton model for simulating dendrite growth and its application in additive manufacturing. *Addit. Manuf.* **47**, 102284 (2021).

10. Yu, Y. *et al.* Impact of fluid flow on the dendrite growth and the formation of new grains in additive manufacturing. *Addit. Manuf.* **55**, 102832 (2022).

11. Liu, D.-R., Wang, S. & Yan, W. Grain structure evolution in transition-mode melting in direct energy deposition. *Mater. Des.* **194**, 108919 (2020).

12. Lian, Y., Lin, S., Yan, W., Liu, W. K. & Wagner, G. J. A parallelized three-dimensional cellular automaton model for grain growth during additive manufacturing. *Comput. Mech.* **61**, 543–558 (2018).

13. Nie, P., Ojo, O. A. & Li, Z. Numerical modeling of microstructure evolution during laser additive manufacturing of a nickel-based superalloy. *Acta Mater.* **77**, 85–95 (2014).

14. W. Kurz; Fisher, D. J. *Fundamental of solidification*. (1984).

15. Lian, Y. *et al.* A cellular automaton finite volume method for microstructure evolution during additive manufacturing. *Mater. Des.* **169**, 107672 (2019).

16. Aziz, M. J. Model for solute redistribution during rapid solidification. *J. Appl. Phys.* **53**, 1158–1168 (1982).

17. Wang, Y., Shi, J. & Liu, Y. Competitive grain growth and dendrite morphology evolution in selective laser melting of Inconel 718 superalloy. *J. Cryst. Growth* **521**, 15–29 (2019).

18. Yi, J. *et al.* Microstructure and mechanical behavior of bright crescent areas in Inconel 718 sample fabricated by selective laser melting. *Mater. Des.* **197**, 109259 (2021).

19. Tang, C., Tan, J. L. & Wong, C. H. A numerical investigation on the physical mechanisms of single track defects in selective laser melting. *Int. J. Heat Mass Transf.* **126**, 957–968 (2018).

20. Hooper, P. A. Melt pool temperature and cooling rates in laser powder bed fusion. *Addit. Manuf.* **22**, 548–559 (2018).

21. Tang, Y. T. *et al.* Alloys-by-design: Application to new superalloys for additive manufacturing. *Acta Mater.* **202**, 417–436 (2021).

22. Ghoussoub, J. N. *et al.* On the Influence of Alloy Composition on the Additive Manufacturability of Ni-Based Superalloys. *Metall. Mater. Trans. A* **53**, 962–983 (2022).
