# Supplementary material for: Solute trapping and non-equilibrium microstructure during rapid solidification of additive manufacturing
Source: Nat Commun. 2023 Dec 2;14:7990. doi: 10.1038/s41467-023-43563-x (PMC10693635; doi:10.1038/s41467-023-43563-x)
Supplement: Supplementary file 3 — Description of Supplementary Files [file 41467_2023_43563_MOESM3_ESM.docx]

**Inventory of Supporting Information**

Manuscript # NCOMMS-23-07132C

Corresponding author name(s): Jun Li, Chinnapat Panwisawas

1. Flat Files

| Item | Filename | Description |
| --- | --- | --- |
| Supplementary Information | revised Supplementary information.docx | Including Supplementary Methods, Results and Discussion, Figures, and References |
| Supplementary Movie | Supplementary Movie 1.avi | Supplementary movie |
| Completed Third Party Rights Table | Copyrights.rar | The copyrights of the adapted figures from the references |

1. Source Data.

| Item | Filename | Description |
| --- | --- | --- |
| Source Data files | Source Data file.rar | The original version of the adapted figures from the cited references |
